# Osteoporosis1Read before the Virginia State Veterinary Medical Association, January 2, 1896.

**Published:** 1896-05

**Authors:** George C. Faville

**Affiliations:** Norfolk, Va.


### OSTEOPOROSIS.[1]

By GEORGE C. FAVILLE, D.V.M.,

NORFOLK, VA.

(Concluded from page 262.)

Replying to my questions, Dr. Harbaugh says:

1. Osteoporosis is met with in the range of my practice to a greater or less extent every year, but in some seasons it appears in enzoötic form, when the number of cases diagnosed increases the average to a great extent. I have only a limited knowledge of the disease elsewhere in the State, but from different persons I learn that it is by no means confined to this vicinity.

2. According to my observations the disease attacks old and young horses alike. In one stable where all the horses were over six years old, nine horses contracted the disease within a period of six months. In another stable where all the animals were young, two two-year-olds and three three-year-olds were markedly diseased, and some both older and younger showed no symptoms whatever.

3. In all my experience the affected animals were stabled. While it is true that some of the animals were turned out to graze or exercise during the day, and that the affected city-horses were out in the air daily at their accustomed work, still all were stabled at night. In every instance the stable was more or less damp, and lacked proper ventilation, drainage, and light.

In no case could I justly find fault with food and water. With the great majority of affected animals the food was the best that could be procured in the market, and, being to a great extent what we know as Western food, it was practically the same food as fed to the thousands of non-affected animals. In the city the water from the hydrants is used, James River water, and is, according to numerous tests by competent chemists, a practically pure drinking-water.

4. I have met with the disease in animals ranging from yearlings to aged horses, but never saw a case in a mule, ass, or hinny. I am firmly convinced that the predisposing cause of

[1] Read before the Virginia State Veterinary Medical Association, January 2, 1896.

the disease is undoubtedly the faulty construction of stables. The site of the stable, and surroundings also, have much to do with the cause. I infer that the want of proper ventilation, sunlight, and drainage in and about stables is the predisposing cause of osteoporosis. I have never seen a case in a stable where the hygienic essentials received attention. That the many badly-constructed stables with little or no ventilation do not always produce the disease proves nothing; the predisposing cause is there, but the exciting cause is wanting.

As to the nature of the proximate cause I am not at present prepared to express an opinion. In this age of microbes one dare not say that such a disease is not due to specific microorganisms, but I am confident that osteoporosis is not a contagious disease. All my observations point to the fact that the disease is never transmitted from one animal to another, and I am equally positive that the affected animal cannot infect a place where he is kept, either directly or indirectly.

5. Treatment cannot possibly be satisfactory if the animal is kept in the stable where he contracted the disease. The first and essential thing to do is to remove the affected animal or animals to desirable quarters. As the conditions which predispose an animal to osteoporosis are such as produce rheumatism, I am not surprised to find rheumatism as a complication in a great majority of my cases, and hence this complication requires special attention. For the rheumatic complication I obtain satisfactory results from the administration of either salol and phenacetin or salicylate of soda and acetanilid. When the rheumatic symptoms are relieved I direct my attention to the deranged functions of nutrition and assimilation. The only medicinal agent I use for this purpose is the precipitated phosphate of lime. It is continued for a long time, as circumstances seem to necessitate. As to food, I always prefer a mixed diet; corn, oats, and bran, fed as a mixture, will better assume essential nutriment than either fed separately. It is scarcely necessary to remark that all food should be of the best quality. Turning affected animals on good pasture in proper season is of undoubted benefit, but they should also have daily allowances of grain.

6. I have not kept a record of all my cases, but I am sure that over 75 per cent. of those treated have made good recoveries. Of those in which the disease is recognized in an early stage, which is always possible in enzoötics, all make good recoveries, when you can have the supervision of the animal until recovery is assured. In such cases even the bones of the head, which have become perceptibly enlarged, regain to a greater or less extent their normal condition. Of course, I occasionally see a case beyond hope, but, as a rule, really bad cases can be very much improved, and often become serviceable work-horses when the unsightly enlarged facial bones unfit them for pleasure purposes.

The length of time required for even a partial restoration is the great drawback when expense is considered. This feature prevents many owners from putting their affected animals under treatment when informed of the nature of the disease, especially when the affected animals are of little value.

*Remarks.* I cannot agree with the eminent authorities who class osteoporosis as a non-inflammatory disease of bone. The ordinary symptoms of inflammation are heat, pain, redness, and swelling, and they are also the symptoms of osteoporosis.

The quantity and quality of the food and water have nothing to do with the cause of osteoporosis directly. Food may possibly become a factor, the result of conditions, but deficiency of lime-salts in water or food does not produce the disease as I find it. The food may be perfect in every respect, but, as the disease is one seriously affecting the functions of nutrition and assimilation, the lime-salts which the food naturally contains are not properly appropriated. Either directly or indirectly the result of the inflammatory action going on in the bones, the lime-salts they contain are converted into soluble salts and re-absorbed, leaving the affected bones in the condition we find them. I am aware that this is a variation of the acid theory, but, to my mind, it accounts for the characteristic phenomena. I do not give phosphate of lime solely for the purpose of furnishing lime-salt to the bones; I give it to correct the mal-nutrition of the bones, as well as of all the fluids and tissues of the body, of which it is a normal constituent.

In all my experience osteoporosis is confined to the horse; I have never diagnosed a case in any other animal. It is by no means a disease of the poor man's stable. The great majority of my cases were in stables where the animals had the benefit of all that money could procure in the form of first-class food. In one stable, which was a brick structure, the horses' stalls were in the rear, the stone-paved floor of which was from seven to fifteen feet below the grade of the alley. The brick wall against the earth was always wet, and, of course, the atmosphere in that part was moist. Nine horses in the stalls ranged along this brick wall developed the disease, but no case developed among the horses standing in stalls on the opposite side of the stable, as the windows and door on this side were exposed to the south, assuring plenty of sunlight, and the outside being lower than the inside there was surface-drainage, and as this row of stalls was exposed to a plentiful supply of outside air ventilation occurred naturally to some extent. When called to examine an affected horse I expressed the opinion that the structure of the stable and want of proper hygienic conditions caused the trouble. The owners took my advice, and converted the floor above into a stable. Since the horses have been kept there no case of the disease has developed, and those among the affected which were treated recovered sufficiently to be able to do hard work for months past. I have never been able to trace the disease to even a suspicion of a hereditary predisposition.

